# Inhibition of the Proliferation of Human Lung Fibroblasts by Prostacyclin Receptor Agonists is Linked to a Sustained cAMP Signal in the Nucleus

**DOI:** 10.3389/fphar.2021.669227

**Published:** 2021-04-29

**Authors:** Maxine J. Roberts, Lauren T. May, Alastair C. Keen, Bonan Liu, Terrance Lam, Steven J. Charlton, Elizabeth M. Rosethorne, Michelle L. Halls

**Affiliations:** ^1^ Cell Signalling Research Group, School of Life Sciences, University of Nottingham, Queen’s Medical Centre, Nottingham, United Kingdom; ^2^ Drug Discovery Biology Theme, Monash Institute of Pharmaceutical Sciences, Monash University, Parkville, Vic, Australia; ^3^ Excellerate Bioscience Ltd., BioCity, Nottingham, United Kingdom

**Keywords:** G protein-coupled receptor, cAMP, prostacyclin receptor, localised signalling, extracellular signal-regulated kinase

## Abstract

Idiopathic pulmonary fibrosis is a chronic and progressive fibrotic lung disease, and current treatments are limited by their side effects. Proliferation of human lung fibroblasts in the pulmonary interstitial tissue is a hallmark of this disease and is driven by prolonged ERK signalling in the nucleus in response to growth factors such as platelet-derived growth factor (PDGF). Agents that increase cAMP have been suggested as alternative therapies, as this second messenger can inhibit the ERK cascade. We previously examined a panel of eight Gα_s_-cAMP-coupled G protein-coupled receptors (GPCRs) endogenously expressed in human lung fibroblasts. Although the cAMP response was important for the anti-fibrotic effects of GPCR agonists, the magnitude of the acute cAMP response was not predictive of anti-fibrotic efficacy. Here we examined the reason for this apparent disconnect by stimulating the Gα_s_-coupled prostacyclin receptor and measuring downstream signalling at a sub-cellular level. MRE-269 and treprostinil caused sustained cAMP signalling in the nucleus and complete inhibition of PDGF-induced nuclear ERK and fibroblast proliferation. In contrast, iloprost caused a transient increase in nuclear cAMP, there was no effect of iloprost on PDGF-induced ERK in the nucleus, and this agonist was much less effective at reversing PDGF-induced proliferation. This suggests that sustained elevation of cAMP in the nucleus is necessary for efficient inhibition of PDGF-induced nuclear ERK and fibroblast proliferation. This is an important first step towards understanding of the signalling events that drive GPCR inhibition of fibrosis.

## Introduction

Idiopathic pulmonary fibrosis (IPF) is a chronic disease of the lung that is characterised by a progressive increase in fibrosis of the interstitial tissue (Raghu et al., 2011). Areas of active alveolar epithelial cells and myofibroblasts co-localise in the lungs of patients with IPF to contribute to disease progression (Uhal et al., 1998). Alveolar epithelial cells trigger the secretion of a range of cytokines/growth factors, such as platelet-derived growth factor (PDGF) and transforming growth factor β (TGF-β), which promotes the migration, proliferation, and differentiation of fibroblasts (Khalil et al., 1996; Pan et al., 2001). Elevated levels of PDGF have been found in bronchoalveolar lavage fluid in animal models of disease (Walsh et al., 1993) and in epithelial cells from the lung of patients with IPF (Antoniades et al., 1990). TGF-β levels are also increased in the idiopathic pulmonary fibrotic lung, especially in alveolar epithelium and macrophages (Khalil et al., 1991; Khalil et al., 1996). In IPF, fibroblasts differentiate into myofibroblasts that persist inappropriately in foci of the fibrotic lung. Accumulation of these myofibroblasts leads to excessive deposition of extracellular matrix, increased tissue stiffness, and scarring, all of which contribute to the progressive loss of lung function (Maher et al., 2007).

Two drugs are currently approved for the treatment of IPF; nintedanib, an intracellular tyrosine kinase inhibitor (Hilberg et al., 2008), and pirfenidone, a pyridine with broad anti-inflammatory/anti-TGF-β activity (Iyer et al., 1999; Iyer et al., 2000). Nintedanib shows an anti-fibrotic effect in lung fibroblasts from patients with IPF via inhibition of the pro-proliferative effects of PDGF (Hostettler et al., 2014). However, ∼25% of clinical trial participants discontinued its use due to severe adverse effects (Richeldi et al., 2011; Richeldi et al., 2014). Pirfenidone reduced TGF-β signalling, and inhibited proliferation and TGF-β-induced differentiation of lung fibroblasts (Conte et al., 2014). Again ∼15% of participants discontinued use of pirfenidone due to severe adverse effects (Noble et al., 2011; King et al., 2014). Growth factors, such as PDGF and TGF-β, mediate their cellular effects via receptor tyrosine kinases. Signalling by receptor tyrosine kinases through the mitogen activated protein kinase (MAPK) signalling cascade is associated with many of the pro-fibrotic responses observed in IPF (Grimminger et al., 2015). Activation of the MAPK pathway is detected in lung samples from patients with fibrosis (Yoshida et al., 2002) and inhibition of the MAPK pathway prevents progression of established fibrosis in the TGF-α mouse model of fibrosis (Madala et al., 2012). The MAPK signalling cascade–Ras-Raf-MEK-ERK1/2–can be interrupted by the cAMP effector, protein kinase A (PKA). PKA can phosphorylate the Raf N-terminus, which prevents the Raf interaction with Ras, and therefore disrupts the MAPK signalling cascade (Sidovar et al., 2000; Dhillon et al., 2002; Dumaz et al., 2002; Light et al., 2002; Dumaz and Marais, 2003). Therapeutic approaches which increase cAMP can therefore inhibit pro-fibrotic process *in vitro* and *in vivo* (Insel et al., 2012; Zuo et al., 2019).

cAMP levels in a cell can be increased by preventing cAMP breakdown, which is mediated by phosphodiesterases (PDEs). The PDE4 inhibitor, roflumilast, is an approved treatment for another chronic lung disease, chronic obstructive pulmonary disease (COPD), which is associated with lung fibrosis (Wedzicha et al., 2016). Alternatively, cAMP levels can be increased by activating the enzyme that produces cAMP, adenylyl cyclase. Adenylyl cyclase is the major downstream effector of many Gα_s_-coupled G protein-coupled receptors (GPCRs). GPCRs are the largest class of membrane receptors and are the targets of ∼35% of approved drug treatments (Sriram and Insel, 2018). A number of Gα_s_-coupled GPCRs have been suggested as potential therapeutic targets for IPF, including prostaglandin E_2_ receptors (Borok et al., 1991; Huang et al., 2008; Huang et al., 2010; Markovic et al., 2017; Sieber et al., 2018), the β_2_-adrenoceptor (Liu et al., 2004; Lamyel et al., 2011; Herrmann et al., 2017) and the prostacyclin receptor (IPR) (Cruz-Gervis et al., 2002; Kohyama et al., 2002; Zhu et al., 2010; Corboz et al., 2018). IPR agonists are used in the treatment of idiopathic pulmonary arterial hypertension (iPAH) (Humbert and Ghofrani, 2016), a progressive disorder characterised by high blood pressure in the arteries of the lungs. This is caused by hyperproliferation of the pulmonary arterial smooth muscle cells, resulting in a progressive remodelling of the pulmonary vasculature (Voelkel et al., 2012). Many IPF patients develop secondary PAH, which is strongly linked to mortality rates (Rivera-Lebron et al., 2013; Klinger, 2016), and dysregulated cell proliferation underlies both of these conditions. The IPR agonist, iloprost, is used clinically for the treatment of PAH. Moreover, an another IPR agonist, treprostinil, has recently completed a phase II/III clinical trial (NCT02630316) for use in PAH that is associated with interstitial lung disease.

We previously investigated whether there was a correlation between the amount of cAMP produced following activation of eight Gα_s_-coupled GPCRs, and the degree to which this same stimulus could inhibit the proliferation and fibroblast to myofibroblast transition (FMT) of human lung fibroblasts (Roberts et al., 2018). Although we found that cAMP can inhibit fibroblast proliferation, there was no correlation between the maximal effect of agonists for cAMP accumulation and the degree of inhibition of fibroblast proliferation or FMT. This was particularly evident for the IPR agonists, MRE-269, treprostinil and iloprost. MRE-269 completely reversed PDGF-induced proliferation and TGFβ-induced FMT but was a partial agonist for cAMP accumulation. Conversely, treprostinil partially inhibited PDGF-induced proliferation and TGFβ-induced FMT, despite generating a maximal cAMP signal. More strikingly, iloprost generated a maximal cAMP signal but was unable to block PDGF-induced fibroblast proliferation. These data suggested a disconnect between activation of cAMP signalling, and inhibition of the proliferation of human lung fibroblasts (Roberts et al., 2018). However, signalling in cells is tightly regulated in both space and time, and spatiotemporal control of signalling is key to the activation of specific cellular responses (Ellisdon and Halls, 2016; Halls, 2019). This has been clearly demonstrated by the formation of location specific cAMP signalling complexes (Tsvetanova and von Zastrow, 2014; Agarwal et al., 2017; Wright et al., 2018; Bastug-Ozel et al., 2019), and the requirement of sustained nuclear ERK activity for both analgesia (Jensen et al., 2017; Jimenez-Vargas et al., 2020) and proliferation (Balmanno and Cook, 1999; Yamamoto et al., 2006). Distinct spatiotemporal activation of cAMP and ERK signals in response to IPR agonists, could explain their differing ability to inhibit cell proliferation.

Here we focused on the IPR agonists, MRE-269, treprostinil and iloprost, to determine whether their relative efficacy to inhibit PDGF-induced proliferation of human lung fibroblasts could be explained by differences in the spatial and temporal control of second messenger signalling. We found that PDGF caused a transient increase in cytosolic ERK activity, which could be effectively blocked by all agonists. In contrast, PDGF caused a sustained increase in nuclear ERK activity, that was blocked by MRE-269 and treprostinil, but not iloprost. This is consistent with the partial inhibition of fibroblast proliferation by iloprost. The inhibition of the PDGF-induced nuclear ERK activity was dependent on cAMP. All three agonists caused a sustained increase in plasma membrane and cytosolic cAMP. However, while MRE-269 and treprostinil caused a sustained increase in nuclear cAMP, iloprost only stimulated a transient increase in nuclear cAMP. This suggests that the spatial and temporal regulation of signalling is key to the inhibition of PDGF-proliferation by IPR agonists. Only agonists that caused a sustained increase in nuclear cAMP could also inhibit the sustained nuclear ERK activity induced by PDGF, and effectively block PDGF-induced proliferation of human lung fibroblasts.

## Materials and Methods

### Ligands

Iloprost and PDGF-BB were purchased from Merck, MRE-269 was purchased from Cayman Chemical and treprostinil was purchased from Tocris.

### Plasmids

CytoEpac2-camps was from M. Lohse (University of Wurzburg, Germany) (Nikolaev et al., 2004), pmEpac2-camps was from D. Cooper (University of Cambridge, United Kingdom) (Wachten et al., 2010) and nucEpac2 in adenovirus (Ad-Epac-NLS) was from R. Harvey (University of Nevada, Reno, United States) (Agarwal et al., 2017). CytoEKAR GFP/RFP (Addgene plasmid 18680) and nucEKAR GFP/RFP (Addgene plasmid 18682) were from K. Svoboda (Harvey et al., 2008).

### Cell Culture and Transfection

Normal human lung fibroblasts from a healthy/non-smoker Caucasian donor (d42105.2) were purchased from Promocell. Human lung fibroblasts were maintained in Dulbecco’s modified Eagle’s medium (DMEM) supplemented with 4.5 g/L D-glucose, L-glutamine, pyruvate, 10 % (v/v) FBS and 25 mM HEPES at 37°C with 5% CO_2_ in a humidified atmosphere.

### Proliferation Assay

Proliferation was measured in duplicate by incorporation of bromodeoxyuridine (BrdU). Human lung fibroblasts were seeded overnight at 4,000 cells/well in 96-well black optically clear ViewPlates, before being starved for 24 h in culture medium devoid of FBS. Increased proliferation was measured after incubation with increasing concentrations of PDGF in DMEM supplemented with 0.1 % (w/v) human serum albumin (HSA) for 24 h. To measure inhibition of PDGF-induced proliferation, cells were co-incubated for 24 h with vehicle control (0.1% v/v DMSO) or IPR ligands and an EC_80_ concentration of PDGF (5.5 ng/ml) in DMEM supplemented with 0.1% (w/v) HSA. Proliferation was measured using the DELFIA BrdU incorporation assay kit according to the manufacturer’s protocol. BrdU fluorescence was measured by a EnVision Multilabel Plate Reader, using the UV (TRF340) excitation filter (340 nm), Europium emission filter (615 nM) and the LANCE/DELFIA D400 dichroic mirror. To account for the inter-assay variation in levels of proliferation in each experiment, data were normalised to the EC_80_ PDGF response.

### cAMP Accumulation Assay

cAMP accumulation was measured in duplicate using the LANCE cAMP accumulation assay (PerkinElmer). Human lung fibroblasts were seeded into 96-well plates and grown to confluency. On the day of the experiment, cells were incubated with vehicle control (0.1% v/v DMSO), IPR ligands, or a positive control (100 μM forskolin) diluted in stimulation buffer [Hanks’ balanced salt solution (HBSS) with 5 mM HEPES, 5.6 mM glucose, 1.3 mM CaCl_2_, 0.1% (w/v) HSA (pH 7.4) and 5 μM rolipram] for 2 h at 37°C. For experiments using the IPR antagonist, RO-1138452, cells were pre-incubated with increasing concentrations of the antagonist for 10 min prior to addition of an EC_80_ concentration of treprostinil (630 nM). To terminate the reaction, the buffer was aspirated, and 50 μL of ice-cold ethanol was added per well and left to evaporate at room temperature overnight. The cell precipitate was resuspended in 75 μL of lysis buffer [5 mM HEPES, 0.3% Tween 20, and 0.1% (w/v) BSA (pH 7.4)], and then, 5 μL was transferred to a 384-well white OptiPlate (PerkinElmer) on ice. After addition of AlexaFluor647-anti-cAMP antibody and Europium-labelled streptavidin with biotinylated cAMP for 2 h, the plate was read using an EnVision Multilabel Plate Reader (PerkinElmer) using LANCE settings. The data were analyzed against a cAMP standard curve using GraphPad Prism. To account for the inter-assay variation in levels of cAMP produced in each experiment, data were normalised to the forskolin response.

### GloSensor cAMP Assay

Human lung fibroblasts were electroporated with the GloSensor cAMP plasmid (Promega) using the 4D-Nucleofector System and P2 primary cell 4D-Nucelofector X Kit S (Lonza). Cells (10^6^) were suspended in Nucleofector Solution containing plasmid DNA (4 μg) before transfer to a Nucleocuvette. Electroporation was performed using programme EN150. Following electroporation, cells were resuspended in 500 μL complete culture media and seeded into a 384-well plate (20,000 cells/well) for 24 h prior to assay.

The GloSensor™ assay was carried out according to the manufacturer’s instructions (Promega). Briefly, media was aspirated, and cells were incubated in CO_2_-independent media containing 4% v/v GloSensor™ cAMP reagent and incubated for 2 h at final experimental temperature of 37°C. Luminescence was measured on a BMG LABTEK ClarioStar plate reader, with 1 read per well every 3 min, over a period of 30 min at time: −0.5 h (prior to addition of ligand, 0 time point), 0 h (30 min time point), 2.5 h (3 h time point) or 23.5 h (24 h time point). Fibroblasts were stimulated with vehicle control (0.1% v/v DMSO), IPR ligands at a supra-maximal concentration (3 μM), or 10 μM forskolin (positive control). Data were analysed by calculating the area under the curve (AUC) over the 30 min measurement period. The AUC is expressed relative to the forskolin response at the same time point. Data are shown as the mean ± standard error of the mean of *n* biological replicates, as stated.

### ERK Phosphorylation Assay

ERK1/2 phosphorylation was measured in duplicate using the AlphaScreen ERK1/2 SureFire protocol (PerkinElmer). Human lung fibroblasts were seeded overnight in clear 96-well plates (15,000 cells/well) then starved for at least 6 h in growth media devoid of FBS. To monitor increases in phosphorylated ERK, fibroblasts were treated with PDGF at concentrations as stated for up to 30 min at 37°C. To assess inhibition of PDGF-induced phosphorylation of ERK, fibroblasts were pre-incubated with vehicle control (0.1% v/v DMSO) or IPR ligands at the stated concentrations for 30 min, before addition of an EC_80_ concentration of PDGF (5.3 ng/ml) for 20 min at 37°C. Phorbol 12,13-dibutyrate (PDBu, 200 nM, 20 min) was used as a positive control in all experiments. Following stimulation, the cell medium was replaced with lysis buffer and agitated at room temperature for 10 min. To detect phosphorylated ERK, cell lysates were mixed with activation buffer, reaction buffer, AlphaScreen acceptor beads, and AlphaScreen donor beads at a 200:50:250:1:1 (v/v/v/v/v) ratio in a 384-well white ProxiPlate. Plates were incubated in the dark for 1 h at 37°C followed by measurement on an EnVision Multilabel Plate Reader (PerkinElmer) using AlphaScreen settings. To account for the inter-assay variation in levels of ERK phosphorylation produced in each experiment, data were normalised to the 20 min PDBu response or the maximal PDGF response, as stated. Data were fit using a Pharmechanics “rise and fall” time course equation (“baseline then rise-and-fall to baseline time course with drift”), which is freely available (https://www.pharmechanics.com/time-course-tool-pack).

### FRET Biosensors: Single Live Cell Signalling

Human lung fibroblasts were electroporated with cytoEpac2, pmEpac2, cytoEKAR or nucEKAR using the 4D-Nucleofector System and P2 primary cell 4D-Nucelofector X Kit S (Lonza). Cells (10^6^) were suspended in Nucleofector Solution containing plasmid DNA (4 μg for the Epac2 sensors or 5 μg for the EKAR sensors) before transfer to a Nucleocuvette. Electroporation was performed using programme EN150. Following electroporation, cells were resuspended in 500 μL complete culture media and seeded into black optically clear 96-well ViewPlates (50,000 cells/well) for 24 h prior to imaging. The nucEpac2 biosensor was transduced into human lung fibroblasts using adenovirus. Cells were seeded at 7,500 cells/well in black optically clear 96-well ViewPlates before infection with approximately 7 MOI Ad-Epac-NLS for 24 h prior to imaging.

Ratiometric FRET imaging was performed as described previously (Halls et al., 2015). We detected changes in cAMP levels using Epac2-camps (Nikolaev et al., 2004) which undergoes a conformational change after cAMP binding to the cAMP-binding domain of Epac2. We used Epac2-camps targeted to the cytosol (Nikolaev et al., 2004), plasma membrane (Wachten et al., 2010) or nucleus (Agarwal et al., 2017). Changes in ERK activity were detected using EKAR (Harvey et al., 2008), which undergoes a conformational change after ERK phosphorylates a target sequence in the biosensor. We used EKAR targeted to the cytosol or nucleus (Harvey et al., 2008).

Fluorescence imaging was performed using a high-content GE Healthcare INCell 2000 Analyzer with a Nikon Plan Fluor ELWD 40× (NA, 0.6) objective and FRET module as previously described (Halls et al., 2015). For CFP-YFP (Epac2) emission ratio analysis, cells were sequentially excited with a CFP filter (430/24) with emission measured with YFP (535/30) and CFP (470/24) filters and a polychroic mirror, optimized for the CFP-YFP filter pair (Quad3). For GFP-RFP (EKAR) emission ratio analysis, cells were sequentially excited with a fluorescein isothiocyanate (FITC) filter (490/20) with emission measured with dsRed (605/52) and FITC (525/36) filters and a polychroic mirror, optimised for the FITC-dsRed filter pair (Quad4).

Cells were imaged every 2–3 min over 30–60 min, with two fields of view captured per well. At the end of every experiment, the same cells were stimulated for 10 min with a positive control to maximally activate the biosensor: 200 nM PDBu for EKAR or 10 μM forskolin and 100 μM IBMX for Epac2. These data were used to confirm functionality of cells at the end of each experiment (Supplementary Figure S1). Data were analyzed using in-house scripts written for the FIJI distribution of ImageJ (Schindelin et al., 2012), as previously described (Halls et al., 2015). Data were expressed as the F-F_0_ (baseline subtracted FRET ratio for each cell). F-F_0_ responses from all cells in each biological replicate were averaged, and data are expressed as the mean ± standard error of the mean of *n* biological replicates, as stated. Data were fit using a Pharmechanics “rise and fall” time course equation (“baseline then rise-and-fall to baseline time course with drift”), which is freely available (https://www.pharmechanics.com/time-course-tool-pack).

Ratiometric pseudocolor images were generated as previously described (Kardash et al., 2011). A multiplication factor of 10 was applied using the Ratio Plus plugin, the Blue Green Red LUT was applied, and the brightness and contrast range was set to the minimum and maximum FRET ratios within the image stack.

## Results

### There is Disconnect Between IPR Agonist Efficacy for Inhibition of Proliferation Compared to Inhibition of ERK Phosphorylation or Increased cAMP

We previously reported that proliferation of human lung fibroblasts in response to PDGF is dependent on ERK1/2 phosphorylation, and that direct stimulation of adenylyl cyclase to increase cAMP could inhibit PDGF-dependent proliferation with no negative impact on cell viability (Roberts et al., 2018). However, when we activated endogenous Gα_s_-coupled GPCRs in human lung fibroblasts, we found that the magnitude of the cAMP response was not predictive of the extent of the inhibitory effect on proliferation (Roberts et al., 2018).

**FIGURE 1:**
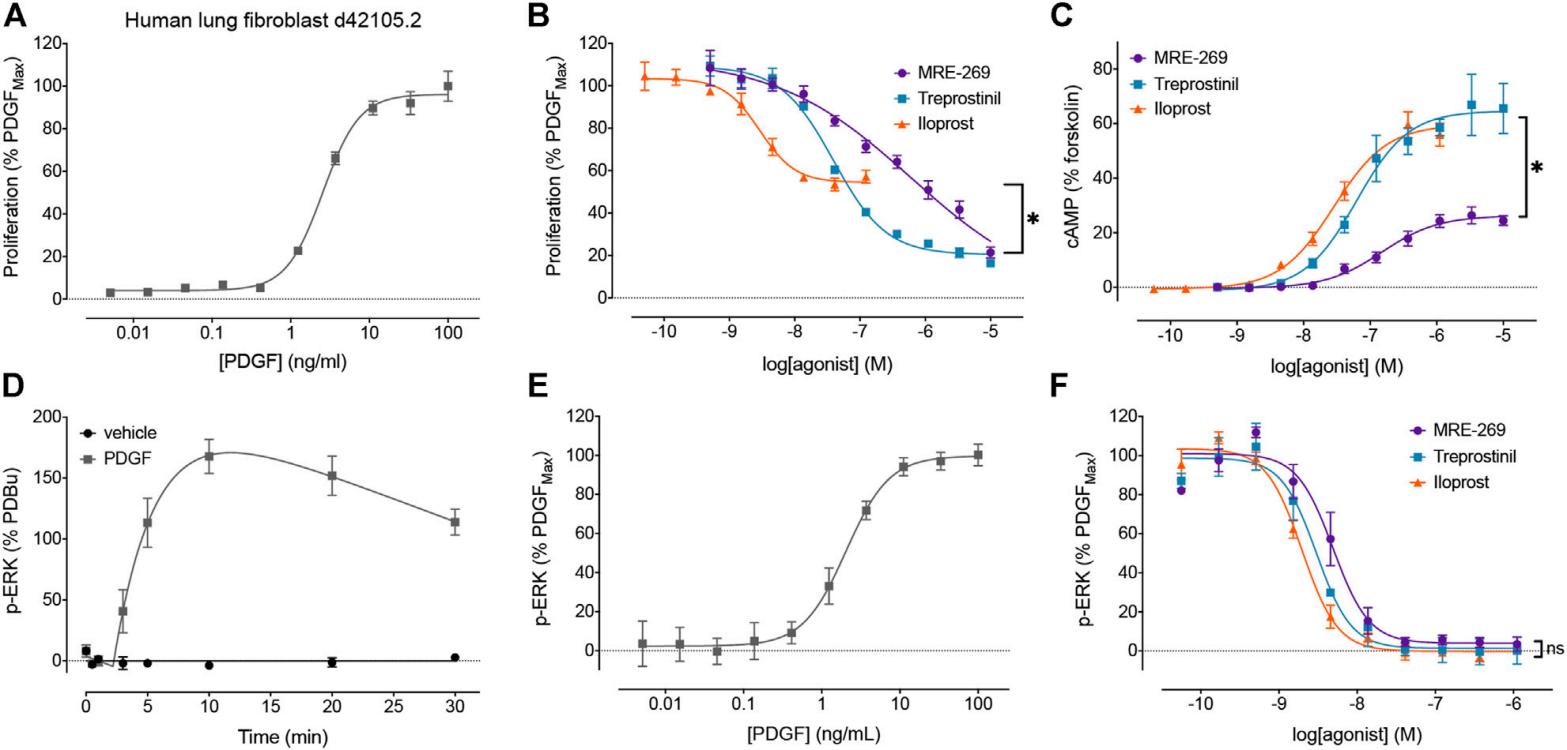
Disconnect between the efficacy of IPR agonists to inhibit PDGF-induced proliferation, and their efficacy for cAMP accumulation and inhibition of PDGF-induced ERK phosphorylation. The relative efficacy of three IPR agonists, MRE-269, treprostinil and iloprost, was determined in human lung fibroblasts from donor d42105.2. **(A)** Proliferation of cells in response to increasing concentrations of PDGF was assessed after 24 h (n = 3). **(B)** Effect of increasing concentrations of MRE-269, treprostinil or iloprost on cell proliferation in response to an EC_80_ concentration of PDGF (5.5 ng/ml) at 24 h (n = 4). **(C)** Effect of increasing concentrations of MRE-269, treprostinil or iloprost on the accumulation of cAMP after 2 h (n = 3). **(D)** Change in ERK phosphorylation after treatment of cells with vehicle (0.01% v/v acetic acid) or a maximal concentration of PDGF (30 ng/ml) over 30 min (n = 3–4). **(E)** Effect of increasing concentrations of PDGF on the phosphorylation of ERK at 20 min (n = 3). **(F)** Effect of increasing concentrations of MRE-269, treprostinil or iloprost on the phosphorylation of ERK in response to an EC_80_ concentration of PDGF (5.5 ng/ml) at 20 min (n = 3). Symbols show the mean, and error bars, standard error of the mean of *n* independent experiments, as stated. For B, *p < 0.05 I_Max_ of iloprost vs the I_Max_ of MRE-269 or treprostinil; for C, *p < 0.05 E_Max_ of MRE-269 vs the E_Max_ of iloprost or treprostinil; for F, no significant (ns) difference in the I_Max_ between all ligands; one-way ANOVA with Tukey’s multiple comparisons test).

We first sought to confirm our previous data using human lung fibroblasts from a different donor (Figure 1). We assessed proliferation by monitoring the incorporation of BrdU, as this method afforded greater sensitivity than cell counting as early as 24 h following stimulation with PDGF (Roberts et al., 2018). PDGF caused a concentration-dependent increase in cell proliferation (Figure 1A). All three IPR agonists caused a concentration-dependent inhibition of cell proliferation induced by an EC_80_ concentration of PDGF (Figure 1B). Both MRE-269 and treprostinil were very effective at blocking PDGF-induced proliferation, reducing the PDGF response to 4.09 ± 13.42 and 17.39 ± 3.45, respectively, compared to control (values are mean ± SEM of the I_Max_, expressed as % of the maximal PDGF response). In contrast, iloprost only partially blocked PDGF-induced proliferation with an I_Max_ of 60.48 ± 2.3%. Treprostinil appeared more effective at inhibiting PDGF-induced proliferation in the lung fibroblasts from donor d42105.2 compared to donors d91019001.2 or d9017402.1 (Roberts et al., 2018). Consistent with our previous data, both iloprost and treprostinil cause a robust, concentration-dependent increase in cAMP accumulation with E_Max_ values of 76.00 ± 12.71 and 86.64 ± 16.99, respectively (values are mean ± SEM of the E_Max_, expressed as % of the forskolin response). In contrast, MRE-269 induced a partial increase in cAMP accumulation, with an E_Max_ of 36.91 ± 3.00 (*p* <0.05 vs the effect of iloprost or treprostinil, one-way ANOVA with Tukey’s multiple comparisons test). Therefore, we again observe a disconnect between efficacy for cAMP accumulation compared to inhibition of PDGF-induced proliferation.

In addition to IPR, treprostinil can bind the Gα_s_-coupled EP2 receptor with sub-micromolar affinity (Whittle et al., 2012), and this receptor is also expressed in human lung fibroblasts (Roberts et al., 2018). Indeed, when we tested the effect of the IPR antagonist, RO-1138452, on the treprostinil-induced cAMP response we observed only ∼60% inhibition of the signal (Supplementary Figure S2). This suggests that the treprostinil-induced cAMP signal is contributed, at least in part, by activation of the EP2 receptor. In contrast, iloprost has sub-micromolar affinity for the Gα_i_-coupled EP1 receptor (Kiriyama et al., 1997) which is expressed at very low levels in human lung fibroblasts (Roberts et al., 2018) and its activation would not increase cAMP. MRE-269 is relatively selective for the IPR (Kuwano et al., 2008). These distinct receptor activation profiles would not explain the disconnect observed.

We have previously shown that inhibition of MEK (upstream of ERK) can prevent PDGF-stimulated proliferation (Roberts et al., 2018). Here, we directly measured the effect of PDGF and IPR agonists on the phosphorylation of ERK1/2 in human lung fibroblasts, using a population-based assay. PDGF caused a large increase in the phosphorylation of ERK that peaked at 10 min (Figure 1D). The PDGF-induced increase in the phosphorylation of ERK was concentration dependent, with an EC_50_ of 2.05 ± 0.12 ng/ml (Figure 1E). All three IPR agonists caused a complete and concentration-dependent inhibition of the phosphorylation of ERK induced by an EC_80_ concentration of PDGF (Figure 1F). This suggests a further disconnect between efficacy for the inhibition of PDGF-induced ERK phosphorylation and cell proliferation.

### Only MRE-269 and Treprostinil, but Not Iloprost, can Inhibit the Sustained Increase in Nuclear ERK Activity Induced by PDGF

To probe the link between cAMP, inhibition of ERK phosphorylation and inhibition of proliferation, we used FRET biosensors targeted to the cytosol or the nucleus to measure second messengers in different cellular locations in real time and in live cells. These FRET biosensors, termed extracellular signal-regulated kinase activity receptors (EKARs), are specific for ERK (Harvey et al., 2008) and measure ERK activity with high temporal resolution and without the need to mechanically disrupt cells (Halls et al., 2016).

**FIGURE 2:**
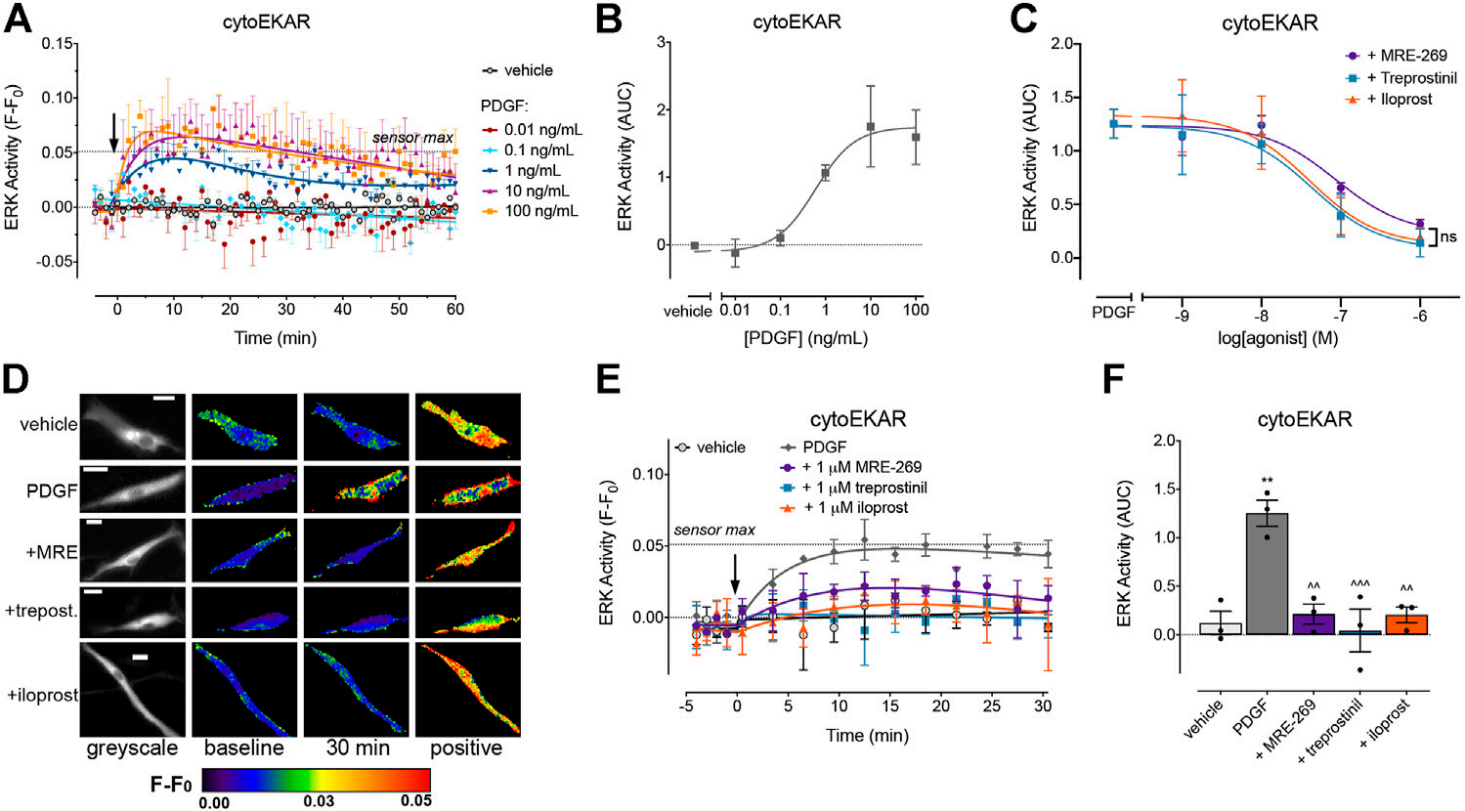
PDGF causes a transient increase in cytosolic ERK activity which is inhibited by MRE-269, treprostinil and iloprost. Cytosolic ERK activity was measured in human lung fibroblasts electroporated with the cytoEKAR FRET biosensor. **(A)** Time course of cytosolic ERK activity in response to vehicle control (0.01% v/v acetic acid) or increasing concentrations of PDGF over 60 min (n = 4). **(B)** Area under the curve (AUC) calculated from A. **(C)** Effect of increasing concentrations of MRE-269, treprostinil or iloprost on cytosolic ERK activity in response to an EC_80_ concentration of PDGF (10 ng/ml, calculated from B) (n = 3). Data are expressed as the 30 min AUC, calculated from time course traces as shown in E. There was no significant difference (ns) in the I_Max_ or pIC_50_ between ligands, one-way ANOVA with Tukey’s multiple comparisons test. **(D)** Representative ratiometric pseudocolour images from C. Images show the effect of 1 μM IPR ligand. Greyscale images are at baseline. Scale bar shows 20 μm. MRE, MRE-269 and treprost., treprostinil. **(E)** Time course of cytosolic ERK activity in response to vehicle control (0.01% v/v acetic acid) or an EC_80_ concentration of PDGF (10 ng/ml) in cells without or with pre-incubation for 30 min with 1 μM IPR ligand (n = 3). **(F)** I_Max_ of the IPR ligands calculated from individual fits of the concentration-response curves. ** *p* < 0.01 vs. vehicle control; ^ ^ *p* < 0.01 and ^ ^ ^ *p* < 0.001 vs. PDGF control; all one-way ANOVA with Dunnett's multiple comparisons test. For time course graphs, data are expressed as the baseline subtracted FRET ratio. Arrow indicates time of vehicle or PDGF addition. Maximal FRET change induced by the positive control (PDBu) is indicated as a dashed line. For AUC graphs, bars show the mean, symbols the individual data points, and error bars show the standard error of the mean.

PDGF caused a concentration-dependent increase in cytosolic ERK activity, which peaked at 10 min, and then declined back towards baseline over the 60 min time course (Figures 2A,B). MRE-269, treprostinil and iloprost caused a concentration-dependent and complete inhibition of the transient PDGF-induced increase in ERK activity (Figures 2C–F). Similar to the ERK phosphorylation assay (Figure 1F), when measured at the single cell level, MRE-269, treprostinil and iloprost were equi-effective for the inhibition of cytosolic ERK activity with no significant difference in the pIC_50_ values of 6.59 ± 0.30, 6.65 ± 0.07 and 6.83 ± 0.29, respectively.

**FIGURE 3:**
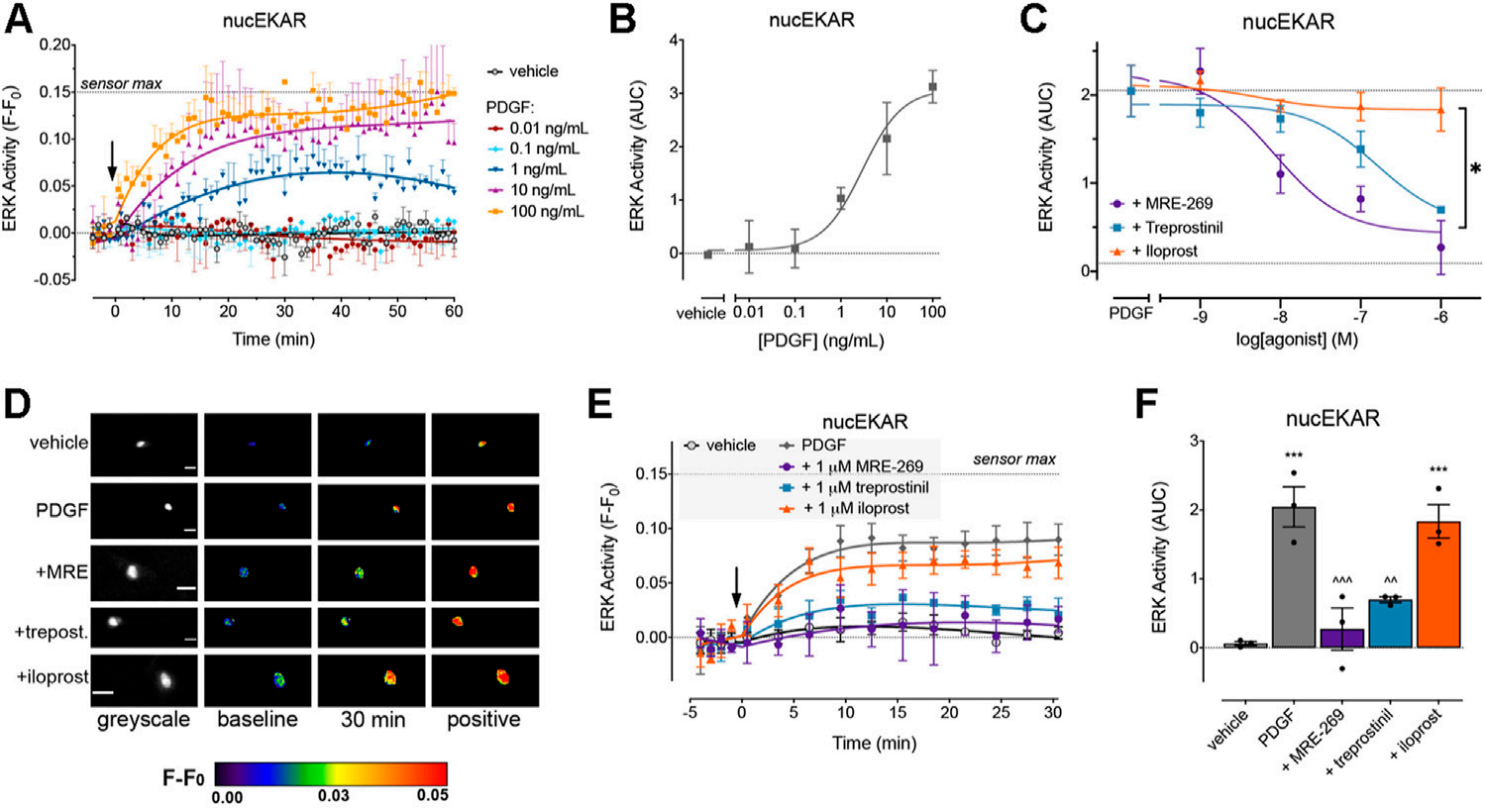
PDGF causes a sustained increase in nuclear ERK activity which is inhibited by MRE-269 and treprostinil but not iloprost. Nuclear ERK activity was measured in human lung fibroblasts electroporated with the nucEKAR FRET biosensor. **(A)** Time course of nuclear ERK activity in response to vehicle control (0.01% v/v acetic acid) or increasing concentrations of PDGF over 60 min (n = 3–4). **(B)** Area under the curve (AUC) calculated from A. **(C)** Effect of increasing concentrations of MRE-269, treprostinil or iloprost on nuclear ERK activity in response to an EC_80_ concentration of PDGF (10 ng/ml, calculated from B) (n = 4). Data are expressed as the 30 min AUC, calculated from time course traces as shown in E. *p < 0.05 I_Max_ of iloprost vs the I_Max_ of MRE-269 or treprostinil, one-way ANOVA with Tukey’s multiple comparisons test. **(D)** Representative ratiometric pseudocolour images from C. Images show the effect of 1 μM IPR ligand. Greyscale images are at baseline. Scale bar shows 20 μm. MRE, MRE-269 and treprost., treprostinil. **(E)** Time course of nuclear ERK activity in response to vehicle control (0.01% v/v acetic acid) or an EC_80_ concentration of PDGF (10 ng/ml) in cells without or with pre-incubation for 30 min with 1 μM IPR ligand (n = 3). **(F)** AUC calculated from E. **p < 0.01 vs. vehicle control; ^∧∧^ *p* < 0.01 and ^∧∧∧^ *p* < 0.001 vs. PDGF alone; one-way ANOVA with Dunnett’s multiple comparisons test. For time course graphs, data are expressed as the baseline subtracted FRET ratio. Arrow indicates time of vehicle or PDGF addition. Maximal FRET change induced by the positive control (PDBu) is indicated as a dashed line. For AUC graphs, bars show the mean, symbols the individual data points, and error bars show the standard error of the mean.

PDGF also caused a concentration-dependent increase in ERK activity within the nucleus. In contrast to the transient cytosolic signal, ERK activity within the nucleus was sustained over the 60 min measurement period (Figures 3A,B). MRE-269 and treprostinil caused a concentration-dependent inhibition of the sustained PDGF-induced increase in ERK activity (Figures 3C–F). In contrast, iloprost was unable to block the PDGF-induced increase in nuclear ERK activity (Figures 3C–F).

While all three ligands could block PDGF-induced changes in cytosolic ERK activity, only MRE-269 and treprostinil were able to inhibit PDGF-induced changes in nuclear ERK activity. The inability of iloprost to block PDGF-induced inhibition of nuclear ERK correlates with the relatively minor impact of iloprost on PDGF-induced cell proliferation.

### IPR-Mediated Inhibition of PDGF-Induced Nuclear ERK is Dependent on cAMP

**FIGURE 4:**
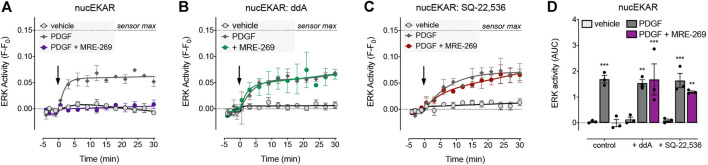
Inhibition of PDGF-stimulated nuclear ERK by MRE-269 is dependent on cAMP. Nuclear ERK activity was measured in human lung fibroblasts electroporated with the nucEKAR FRET biosensor. Cells were stimulated with vehicle control (0.01% v/v acetic acid) or an EC_80_ concentration of PDGF (10 ng/ml) with or without pre-incubation with 1 μM MRE-269 (n = 3). **(A)** No inhibitor treatment. **(B)** Pre-treatment with the adenylyl cyclase inhibitor, dideoxyadenosine (ddA, 30 μM). **(C)** Pre-treatment with the adenylyl cyclase inhibitor, SQ-22,536 (30 μM). **(D)** 30 min AUC calculated from A-C. **p < 0.01 and ***p < 0.001 vs. vehicle control, two-way ANOVA with Dunnett’s multiple comparisons test.

The cAMP-PKA pathway can inhibit ERK signalling via phosphorylation of Raf (Sidovar et al., 2000; Dhillon et al., 2002; Dumaz et al., 2002; Light et al., 2002; Dumaz and Marais, 2003) or by transcriptional up-regulation of Dual Specificity Phosphatases (DUSPs) (Burgun et al., 2000; Stork and Schmitt, 2002). To determine whether the IPR-mediated inhibition of PDGF-induced nuclear ERK was dependent on cAMP, we assessed the effect of two adenylyl cyclase inhibitors, dideoxyadenosine (ddA) and SQ-22,536 (Figure 4). We chose to focus on the MRE-269-mediated inhibition of PDGF-induced nuclear ERK activity, as this provided the largest experimental window with which to assess the effect of adenylyl cyclase inhibition (Figures 3A–C).

There was no effect of ddA on basal levels of ERK activity in the nucleus, nor on the PDGF-induced increase in nuclear ERK activity. However, ddA completely reversed the inhibition of PDGF-induced nuclear ERK activity by MRE-269 (Figures 4A,B,D). Similarly, there was no effect of SQ-22,536 on basal or PDGF-induced nuclear ERK activity, but SQ-22,536 completely reversed the inhibition of PDGF-induced nuclear ERK in response to MRE-269 (Figures 4A,C,D). This suggests that the IPR inhibits PDGF-induced nuclear ERK (and therefore cell proliferation) in a cAMP dependent manner.

### Only MRE-269 and Treprostinil, but not Iloprost, Caused a Sustained Increase in Nuclear cAMP

**FIGURE 5:**
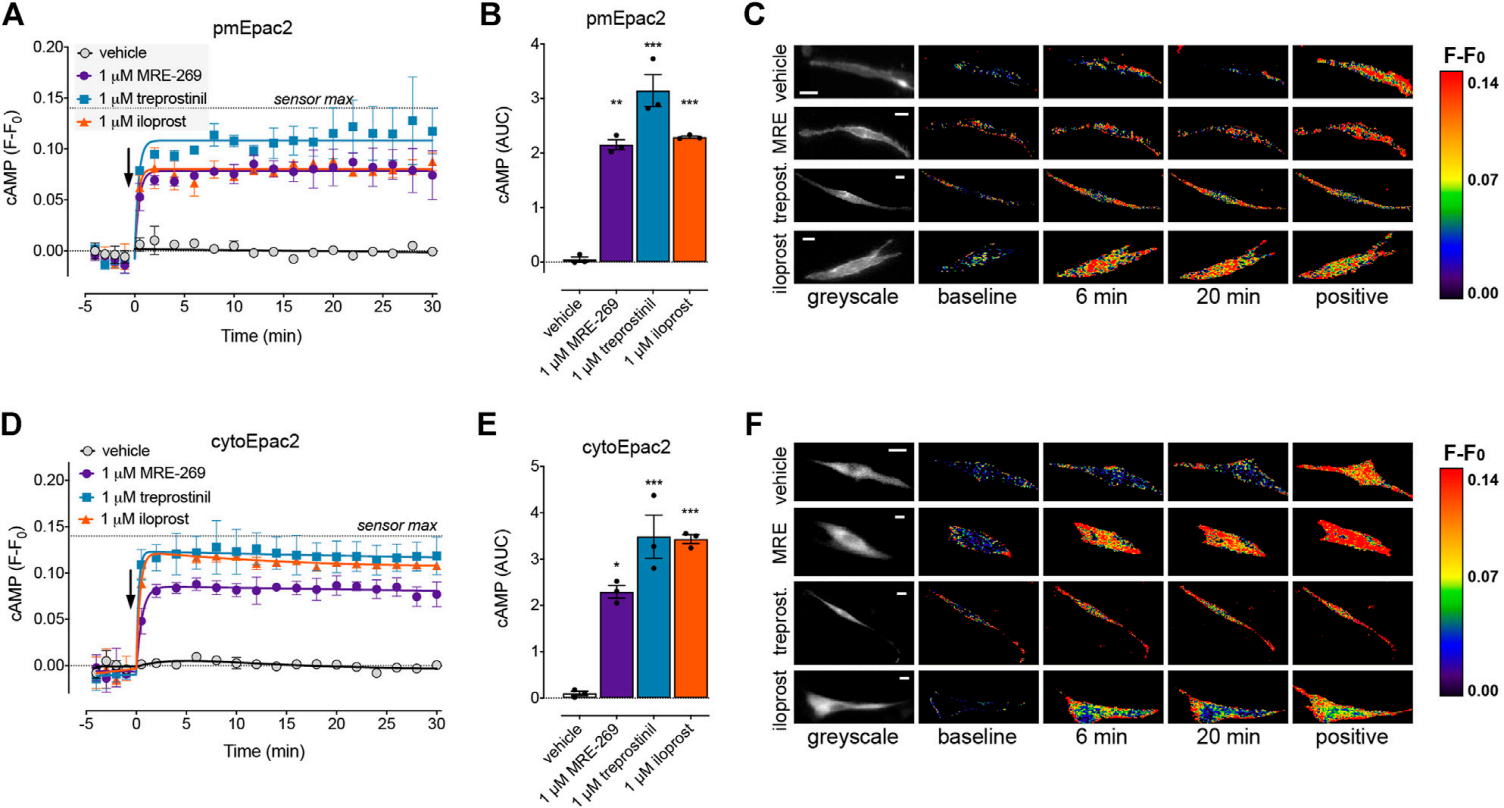
MRE-269, treprostinil, and iloprost all cause sustained increases in plasma membrane and cytosolic cAMP. cAMP was measured at the plasma membrane or in the cytosol of human lung fibroblasts electroporated with the pmEpac2 or cytoEpac2 FRET biosensors, respectively. Cells were stimulated with vehicle control (0.1% v/v DMSO) or a maximal concentration of MRE-269, treprostinil or iloprost (all 1 μM) (n = 3). **(A)** Time course of cAMP at the plasma membrane. **(B)** AUC calculated from A. **(C)** Representative ratiometric pseudocolour images from A. **(D)** Time course of cAMP in the cytosol. **(E)** AUC calculated from D. **(F)** Representative ratiometric pseudocolour images from D. For time course graphs, symbols show the mean and error bars, standard error of the mean. The arrow indicates addition of vehicle or IPR agonist. Maximal FRET change induced by the positive control (forskolin and IBMX) is indicated as a dashed line. For AUC graphs, bars show the mean, symbols the individual data points, and error bars show the standard error of the mean. *p < 0.05, **p < 0.01 and ***p < 0.001 vs. vehicle control, one-way ANOVA with Dunnett’s multiple comparisons test. For ratiometric images, scale bar shows 20 μm. MRE, MRE-269 and treprost., treprostinil.

**FIGURE 6:**
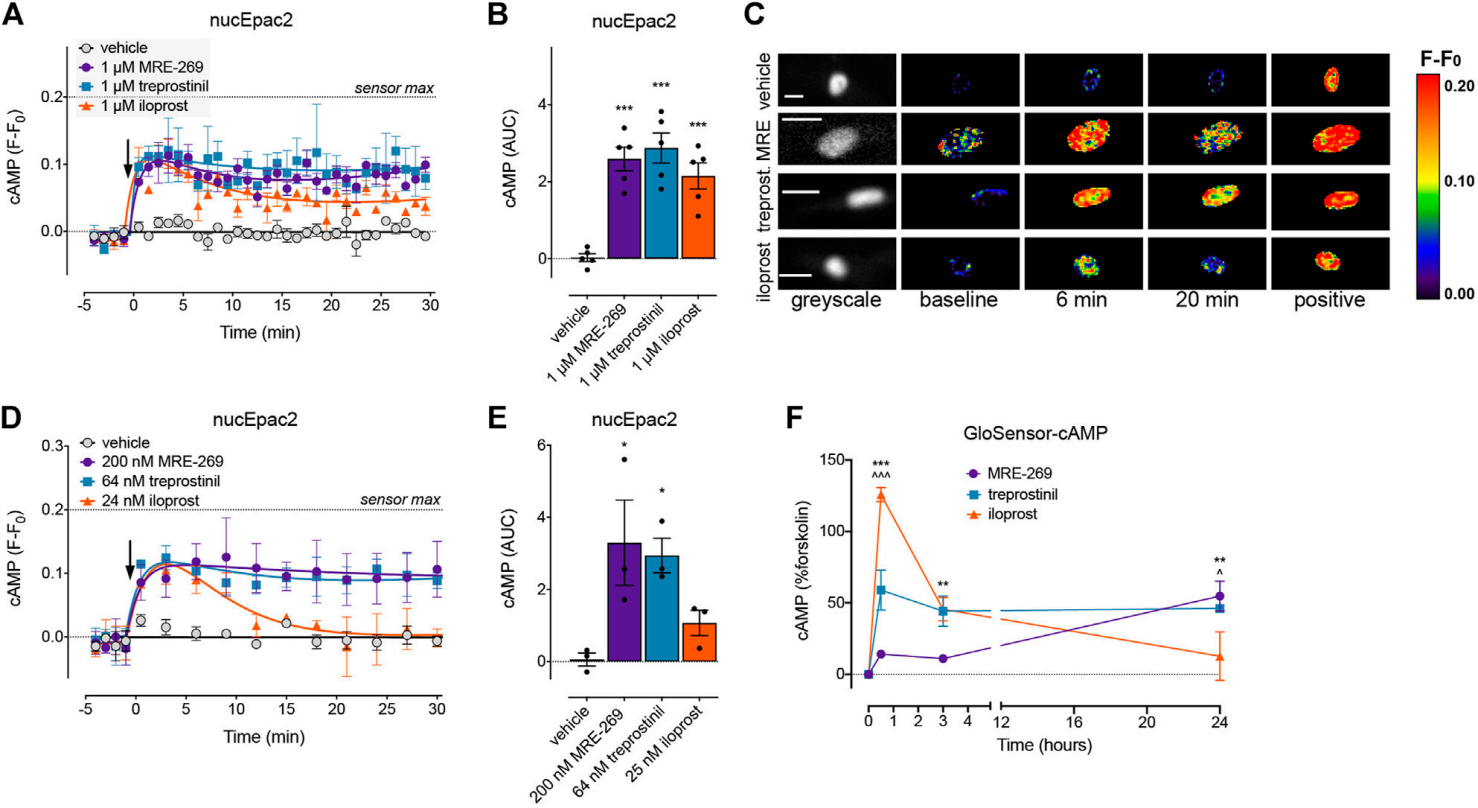
MRE-269 and treprostinil, but not iloprost, cause sustained increases in nuclear cAMP. cAMP was measured in the nucleus of human lung fibroblasts transduced with the nucEpac2 FRET biosensor. Cells were stimulated with vehicle control (0.1% v/v DMSO) or a maximal concentration of MRE-269, treprostinil or iloprost (all 1 μM) (n = 5). **(A)** Time course of cAMP in the nucleus. **(B)** AUC calculated from A. **(C)** Representative ratiometric pseudocolour images from A. Cells were stimulated with vehicle control (0.1% v/v DMSO) or an EC_50_ concentration of MRE-269 (200 nM), treprostinil (64 nM) or iloprost (25 nM) (n = 3). **(D)** Time course of cAMP in the nucleus. (**E**) AUC calculated from D. The arrow indicates addition of vehicle or IPR agonist at time 0. Maximal FRET change induced by the positive control (forskolin and IBMX) is indicated as a dashed line. **(F)** Time course of cAMP over 24 h in response to a maximal concentration of MRE-269, treprostinil or iloprost (n = 3). For time course graphs, symbols show the mean and error bars, standard error of the mean. For AUC graphs, bars show the mean, symbols the individual data points, and error bars show the standard error of the mean. For AUC graphs, *p < 0.05 and ***p < 0.001 vs. vehicle control, one-way ANOVA with Dunnett’s multiple comparisons test. For GloSensor time course graphs, ** *p* < 0.01 and *** *p* < 0.001 for MRE-269 vs. iloprost, ^ *p* < 0.05 and ^ ^ ^ *p* < 0.001 for treprostinil vs. iloprost, two-way ANOVA with Tukey's multiple comparisons test. For ratiometric images, scale bar shows 20 μm. MRE, MRE-269 and treprost., treprostinil.

To determine if there was a difference in the spatial or temporal profile of cAMP signals in response to activation of the IPR by MRE-269, treprostinil or iloprost, we used cAMP FRET biosensors targeted to the plasma membrane, cytosol or the nucleus (Figures 5, 6). The ligands were used at concentrations that induced an EC_50_ or E_Max_ cAMP response, as determined by a whole cell assay (Figure 1C).

At EC_50_ and E_Max_ concentrations, all three ligands caused an equivalent and sustained increase in plasma membrane cAMP over a 30 min time course (Figures 5A–C, Supplementary Figures S3A,B). When cAMP was measured in the cytosol, at EC_50_ and E_Max_ concentrations all three ligands again caused an equivalent and sustained increase in cAMP (Figures 5D–F, Supplementary Figures S3C,D). In the nucleus, at EC_50_ and E_Max_ concentrations both MRE-269 and treprostinil caused a sustained and equivalent increase in cAMP (Figures 6A–E). In contrast, an EC_50_ and E_Max_ concentration of iloprost caused a transient increase in nuclear cAMP which reached a peak by 1 min and declined back to a plateau above baseline (E_Max_) or at baseline (EC_50_) by 20 min (Figures 6A–E). The temporal distinction in the nuclear cAMP response to MRE-269 and treprostinil (sustained) compared to iloprost (transient) was maintained even at EC_10_ concentrations of ligand (Supplementary Figure S4A).

The distinct temporal profiles of nuclear cAMP were not linked to differential IPR endocytosis in response to the three ligands. Inhibition of clathrin-dependent endocytosis (PitStop2) or dynamin-dependent endocytosis (Dyngo4a) had no effect on the nuclear cAMP response to MRE-269, treprostinil or iloprost (Supplementary Figure S4). Finally, we measured cAMP in the human lung fibroblasts following stimulation with the IPR agonists for up to 24 h–the time at which we measured proliferation. We found that MRE-269 and treprostinil caused a sustained cAMP signal up to 24 h (Figure 6F). In contrast, iloprost caused a more transient increase in cAMP, which had returned to baseline by 24 h (Figure 6F). This mimics the temporal profile detected in the nucleus over an acute time frame.

Therefore, effective inhibition of PDGF-induced proliferation by the IPR requires a sustained inhibition of nuclear ERK, and a sustained activation of nuclear cAMP.

## Discussion

**FIGURE 7:**
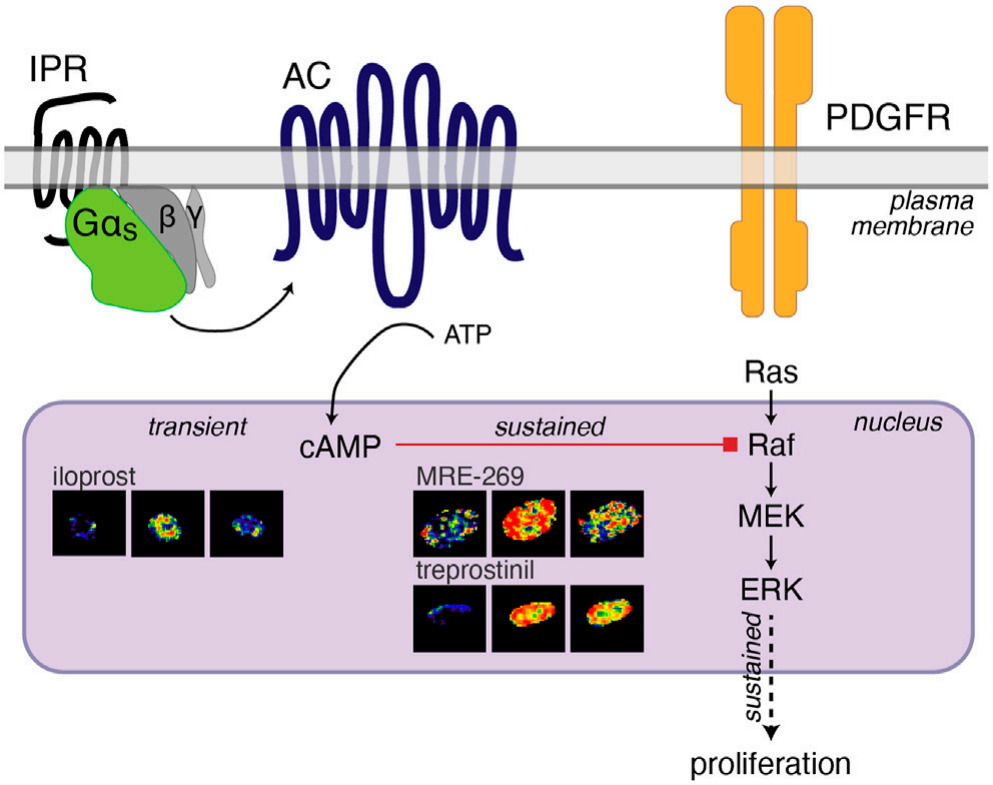
Cartoon of IPR-mediated inhibition of PDGF-induced proliferation. PDGF activation of the PDGF receptor (PDGFR) initiates the Ras-Raf-MEK signalling cascade to increase the phosphorylation of ERK1/2. ERK phosphorylation in the nucleus is sustained over 60 min and sustained nuclear ERK is linked to proliferation (Balmanno and Cook, 1999; Yamamoto et al., 2006). Activation of the IPR by MRE-269 or treprostinil in human lung fibroblasts from donor d42105.2, causes Gα_s_ activation of adenylyl cyclase (AC) to cause a sustained increase in cAMP in the nucleus over 60 min. This sustained elevation of nuclear cAMP is required for the complete inhibition of PDGF-induced ERK and proliferation and is likely mediated by PKA-inhibition of Raf (Sidovar et al., 2000; Dhillon et al., 2002; Dumaz et al., 2002; Light et al., 2002; Dumaz and Marais, 2003). In contrast, iloprost causes a transient increase in cAMP in the nucleus, no inhibition of nuclear ERK and only partially blocks PDGF-induced proliferation.

Therapies that cause an increase in cAMP have been proposed as treatments for lung fibrosis. In human lung fibroblasts we have found that cAMP can inhibit the sustained nuclear ERK response to PDGF (Figure 4), and that ERK is required for PDGF-induced cell proliferation (Roberts et al., 2018). However, we have also previously described a disconnect between the efficacy of Gα_s_-coupled GPCR agonists for inhibiting proliferation and FMT of human lung fibroblasts and the efficacy of the same agonists for cAMP accumulation (Roberts et al., 2018). Here, we report the same disconnect for three IPR agonists–MRE-269 can cause complete inhibition of PDGF-induced proliferation, but is only a partial agonist for cAMP production; treprostinil also causes complete inhibition of PDGF-induced proliferation but is a full agonist for cAMP production; whereas iloprost causes only partial inhibition of PDGF-induced proliferation, but is a full agonist for cAMP production. We now find that this apparent disconnect is linked to the sub-cellular location and duration of the cAMP signal. Both MRE-269 and treprostinil cause a sustained increase in cAMP at the plasma membrane, in the cytosol and in the nucleus of human lung fibroblasts, that is maintained for up to 24 h. In contrast, iloprost causes a sustained increase in plasma membrane and cytosolic cAMP but only a transient increase in nuclear cAMP. No cAMP remains after iloprost stimulation at 24 h. This transient increase in nuclear cAMP in response to iloprost appears insufficient to inhibit the PDGF-induced increase in nuclear ERK and is associated with only partial inhibition of PDGF-induced proliferation (Figure 7).

It has been a long-held view that GPCR signalling occurs on the plasma membrane only, with initial agonist-receptor stimulation often being rapid and short lived, mainly due to desensitisation of the receptor through internalisation. The development of novel methods for visualising proximal GPCR signalling in living cells has been instrumental in the discovery that many GPCRs, including the thyroid stimulating hormone receptor, the parathyroid hormone receptor, the β_1_-and β_2_-adrenoceptors, the neurokinin 1 receptor and the delta opioid receptor, continue signalling via G proteins after internalisation (Calebiro et al., 2009; Ferrandon et al., 2009; Irannejad et al., 2013; Irannejad et al., 2017; Jensen et al., 2017; Jimenez-Vargas et al., 2020). The prolonged duration of second messenger signalling from intracellular locations controls unique cellular responses (Tsvetanova and von Zastrow, 2014; Jensen et al., 2017; Jimenez-Vargas et al., 2020). Therefore, the duration and location of GPCR signalling has a key role in the end biological outcome. Localised GPCR signalling at intracellular locations can be initiated following receptor internalisation (as discussed above), but also from receptors that remain at the plasma membrane. For example, activation of the mu-opioid receptor with DAMGO causes a lateral translocation of the receptor within the plasma membrane, which is associated with an increase in nuclear ERK activity (Halls et al., 2016). This occurs prior to receptor internalisation. It is also clear the localised signals can be initiated from GPCRs that occur on intracellular membranes (and that have not been internalised from the cell surface) (Irannejad et al., 2017). Activation of such intracellular GPCRs is dependent on the lipophilicity of their ligands and therefore whether the ligands can cross the plasma membrane (Irannejad et al., 2017). Here, we find no effect of endocytosis inhibitors on the nuclear cAMP response to the three IPR ligands, suggesting that IPR localised signalling is not dependent on receptor internalisation. MRE-269 is more lipophilic than treprostinil and iloprost (calculated Log *p* using Chemistry Development Kit software (Willighagen et al., 2017): MRE-269 5.3, treprostinil 4.1 and iloprost 3.1), but whether this is sufficient to facilitate differential partitioning into the cell by the agonists is unknown. Future studies using, for example, fluorescently labelled ligands (Comeo et al., 2021), fluorescently tagged nanobodies (Irannejad et al., 2013) or fluorescently tagged miniG proteins (Wan et al., 2018), will be required to definitively determine the origin and location of the receptor pool that controls nuclear signalling.

There is a clear connection between increased nuclear ERK activity and cellular proliferation (Balmanno and Cook, 1999; Yamamoto et al., 2006). Using inhibitors of adenylyl cyclase, we found that cAMP was important for inhibiting nuclear ERK activity (Figure 4). However, whether that cAMP originates from the nucleus or diffuses from other intracellular locations is as yet unknown. Spatial restriction of intracellular cAMP is important for the regulation of biological responses in highly organised cardiomyocyte cells (Schleicher and Zaccolo, 2018; Dikolayev et al., 2019; Halls, 2019). However, the relative importance of spatial control is likely to vary between different cell types and physiological processes. For example, cardiomyocytes need to have very rapid activation and deactivation mechanisms, so cAMP may need to be more tightly regulated to achieve this. In contrast, fibroblast proliferation occurs over a much longer timescale of many hours to days. Therefore, tight spatial regulation of cAMP may not be as important. Our data suggest that this may be the case in human lung fibroblasts–while all three agonists could increase cAMP to the same apparent level in the different cellular compartments, only iloprost was unable to cause a sustained elevation of cAMP in the nucleus. The cAMP response to iloprost in this compartment was instead transient, which may suggest that the temporal profile of second messenger signalling is more important than its spatial restriction. This temporal difference also likely explains the inability of iloprost-induced nuclear cAMP to inhibit PDGF-induced nuclear ERK. The fibroblasts were pre-incubated with the IPR agonists for 30 min prior to challenge with PDGF. At this point, there is no longer any cAMP in the nucleus in cells treated with iloprost (and therefore no mechanism to inhibit the PDGF-induced ERK signal). In contrast, nuclear cAMP in fibroblasts treated with MRE-269 and treprostinil remains elevated, facilitating inhibition of PDGF-induced ERK activity. Selecting the relevant time point for second messenger signalling is therefore important in order to understand time-dependent cellular responses. Measuring second messenger levels after ligand stimulation at acute time points, e.g. 20–30 min, should provide an accurate prediction of efficacy for acute physiological responses, such as vasodilation. However, for cellular responses that occur on a longer time scale, such as proliferation, measuring second messenger responses at much later time points could ensure a more accurate prediction.

While this study does suggest a link between the temporal signature of cAMP signalling in the nucleus, and the ability of IPR agonists to cause efficient inhibition of nuclear ERK and cellular proliferation, this is likely not the complete picture. Here we measured proximal signalling mediators (cAMP and ERK) for up to 90 min post-IPR stimulation (ERK: 30 min pre-treatment with IPR agonists, 1 h measurement of signalling in response to PDGF), and then compared this to the effect on proliferation after a 24 h stimulation with both IPR agonist and PDGF. Many studies on biased signalling have clearly demonstrated the importance of measuring signals at the same timepoint when comparing the relative effects of agonists (Kenakin and Christopoulos, 2013; Klein Herenbrink et al., 2016; Grundmann and Kostenis, 2017). Indeed, when we measured cAMP in response to the IPR agonists for up to 24 h, we found elevated cAMP in response to both MRE-269 and treprostinil, but not iloprost. If the duration of the cAMP signal in the nucleus is key to the ability of IPR agonists to inhibit cellular proliferation, this needs to be examined in more detail over longer time scales. This is particularly important as cellular events that take place over hours to days can also be complicated by changes at the level of the receptor (e.g. desensitisation) and at the level of gene transcription. In addition, iloprost was unable to affect nuclear ERK in response to PDGF, but still caused a partial inhibition of cellular proliferation – suggesting that inhibition of ERK by cAMP in the nucleus is not the only pathway by which IPR agonists can inhibit fibroblast proliferation. IPR agonists including treprostinil and iloprost can also act via the PPARγ nuclear hormone receptor to affect transcription in peripheral pulmonary arterial smooth muscle cells from the lungs of patients with iPAH (Falcetti et al., 2007; Falcetti et al., 2010). This could account for the partial inhibition of cellular proliferation by iloprost, which occurs independently of nuclear cAMP.

Numerous studies have highlighted the potential for cAMP as an inhibitor of fibrosis, with agonists that target Gα_s_-coupled GPCRs such as the β_2_-adrenoceptor, prostaglandin receptors, and the IPR, inhibiting fibrotic processes including fibroblast proliferation and differentiation (Huang et al., 2007; Herrmann et al., 2017; Lambers et al., 2018). However, there is not a clear relationship between efficacy for cAMP accumulation and inhibition of fibrosis (Roberts et al., 2018). By measuring cAMP and ERK signalling at a sub-cellular level, we have now identified a link between sustained increases in cAMP in the nucleus, the inhibition of nuclear ERK and strong inhibition of cellular proliferation. This is an important first step in identifying the type of cAMP signal which is associated with efficient inhibition of proliferation following GPCR activation. Future studies would need to identify the precise mechanism of action underlying differential inhibition of proliferation by IPR agonists. This may include searching for an intracellular receptor pool using cell permeable fluorescent ligands, and desensitisation studies of the endogenous IPR in human lung fibroblasts. Examination of RNA microarray data from a meta-analysis of four studies (Plantier et al., 2016) shows that there appears to be no change in gene expression of the key signalling mediators in fibroblasts from patients with IPF (Supplementary Figure S5). However, future studies need to confirm whether the spatial and temporal organisation of signalling identified here is maintained during disease. A better understanding of the signalling events that drive GPCR inhibition of fibrosis will aid the development of more efficacious treatments for fibrotic diseases such as idiopathic pulmonary fibrosis.

## Data Availability

The raw data supporting the conclusions of this article will be made available by the authors, without undue reservation.
