# Supplementary material for: Inhibition of the Proliferation of Human Lung Fibroblasts by Prostacyclin Receptor Agonists is Linked to a Sustained cAMP Signal in the Nucleus
Source: Front Pharmacol. 2021 Apr 29;12:669227. doi: 10.3389/fphar.2021.669227 (PMC8116805; doi:10.3389/fphar.2021.669227)
Supplement: Supplementary file 1 [file DataSheet1.PDF]

frontiers

Supplementary Material

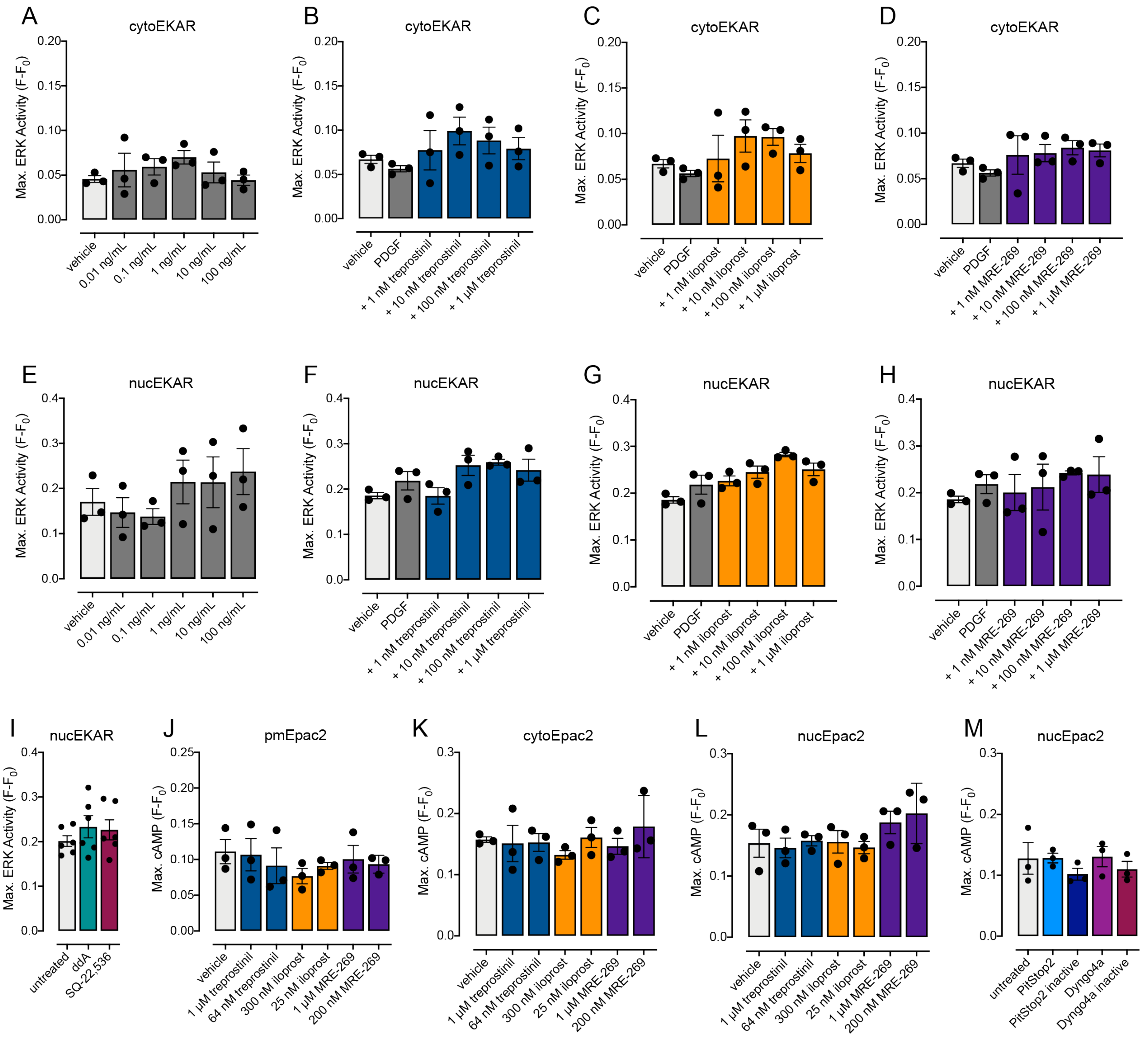

Supplementary Figure 1. Treatment of human lung fibroblasts with PDGF, IPR agonists, AC inhibitors or inhibitors of endocytosis did not affect signalling capacity. The maximal signalling capacity of the human lung fibroblasts was assessed at the conclusion of each FRET biosensor experiment using cytoEKAR (A-D, corresponding to Figure 2), nucEKAR (E-I, corresponding to Figures 3 and 4), pmEpac2 (J, corresponding to Figure 5), cytoEpac2 (K, corresponding to Figure 5) or nucEpac2 (L-M, corresponding to Figure 5 and Figure 6) to confirm cell functionality. Data are expressed as the increase in signalling induced by a positive control (PDBu for ERK, forskolin and IBMX for cAMP). Bars show the mean, symbols the individual data points, and error bars show the standard error of the mean.

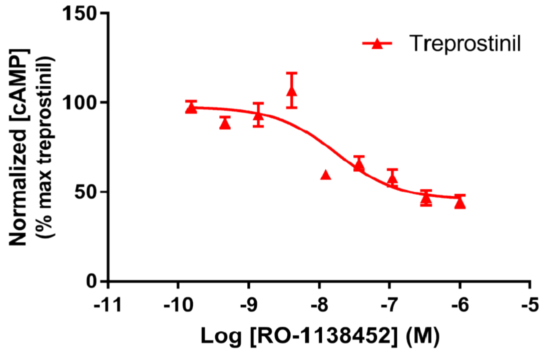

Supplementary Figure 2. Treprostinil also activates EP2 receptors to increase cAMP in human lung fibroblasts. Effect of increasing concentrations of the IPR antagonist, RO-1138452, on the accumulation of cAMP in cell populations treated with an EC_{80} concentration (630 nM) of treprostinil (n=3). Symbols show the mean, and error bars show the standard error of the mean.

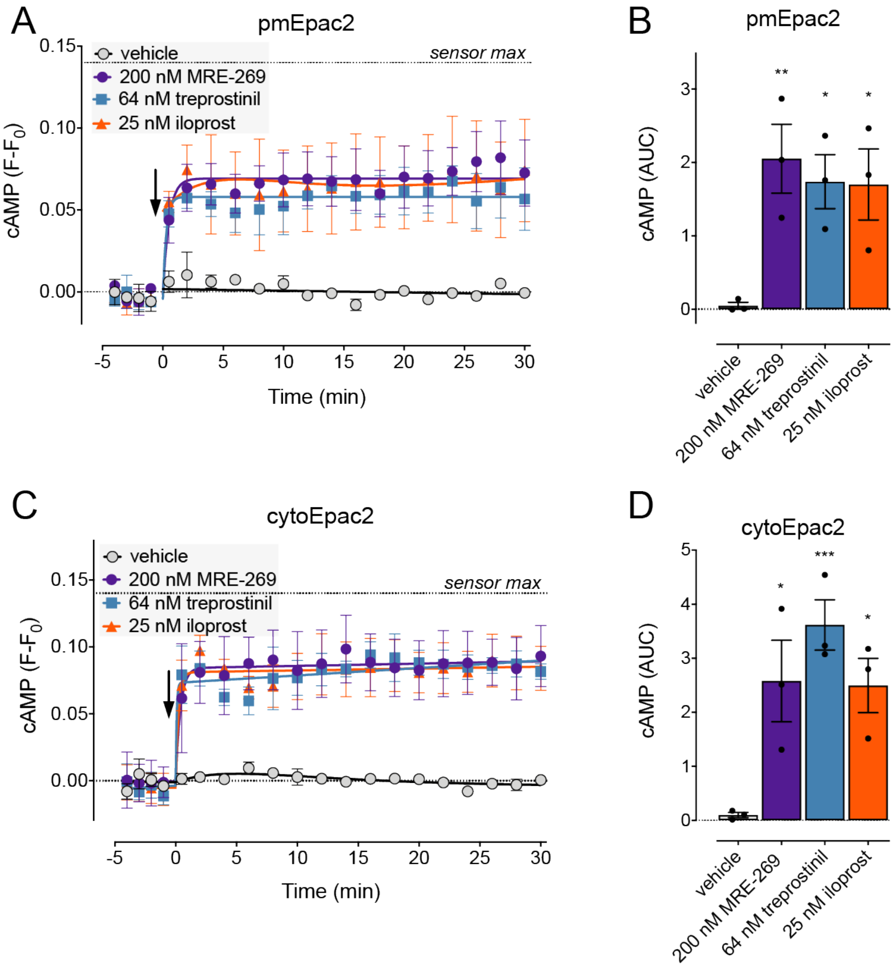

Supplementary Figure 3. EC_{50} concentrations of IPR agonists have equivalent effects on cAMP at the plasma membrane and in the cytosol of human lung fibroblasts. cAMP was measured at the plasma membrane or in the cytosol of human lung fibroblasts electroporated with the pmEpac2 or cytoEpac2 FRET biosensors, respectively. Cells were stimulated with vehicle control (0.1% v/v DMSO) or an EC_{50} concentration of MRE-269 (200 nM), treprostinil (64 nM) or iloprost (25 nM) (n=3). (A) Time course of cAMP at the plasma membrane. (B) AUC calculated from A. (C) Time course of cAMP in the cytosol. (D) AUC calculated from C. For time course graphs, symbols show the mean and error bars, standard error of the mean. The arrow indicates addition of vehicle or IPR agonist. Maximal FRET change induced by the positive control (forskolin and IBMX) is indicated as a dashed line. For AUC graphs, bars show the mean, symbols the individual data points, and error bars show the standard error of the mean. * $p<0.05$, ** $p<0.01$ and *** $p<0.001$ versus vehicle control, one-way ANOVA with Dunnett's multiple comparisons test.

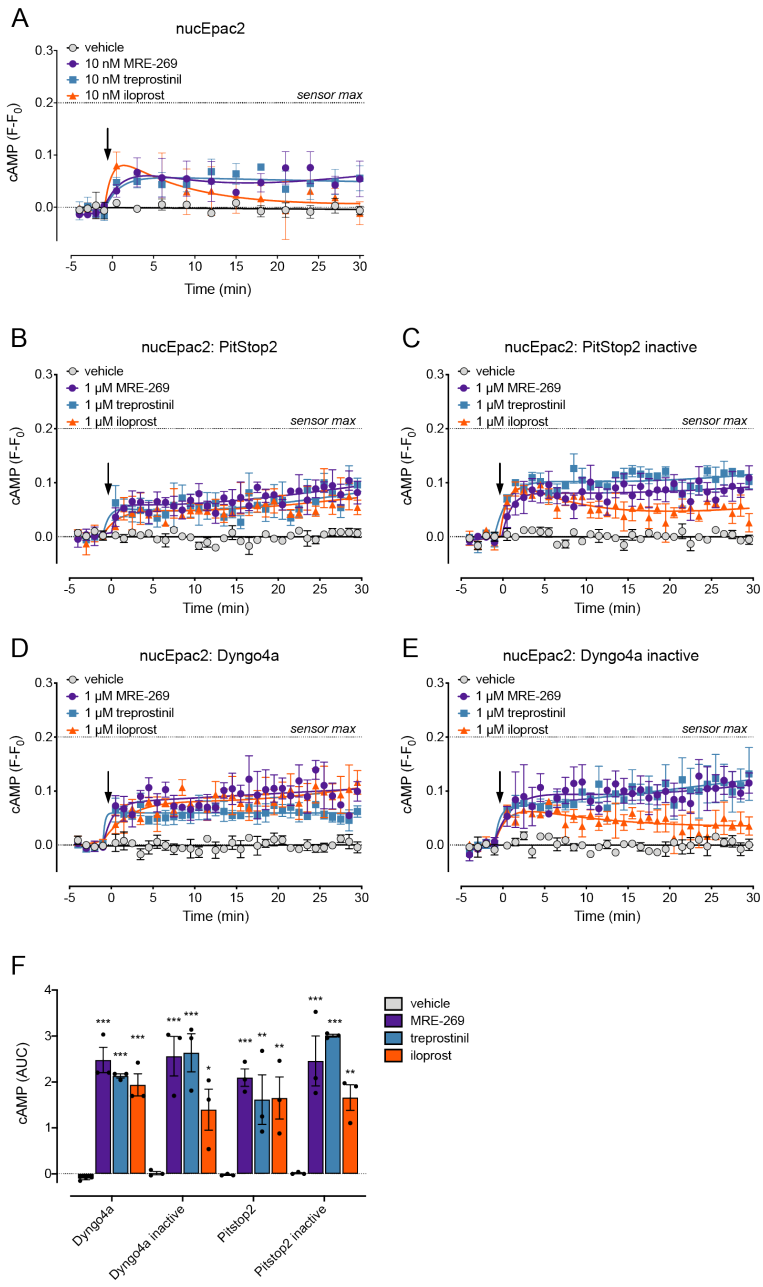

Supplementary Figure 4. Inhibition of endocytosis has no effect on the nuclear cAMP response to IPR agonists in human lung fibroblasts. cAMP was measured in the nucleus of human lung fibroblasts transduced with the nucEpac2 FRET biosensor. (A) Temporal profiles of the IPR agonists were maintained in response to an EC_{10} concentration of MRE-269, treprostinil and iloprost (all 10 nM) (n=3). To assess any role of IPR endocytosis, cells were pre-treated with inhibitors for 30 min prior to stimulation with vehicle control (0.1% v/v DMSO) or a maximal concentration of MRE-269, treprostinil or iloprost (all 1 µM) (n=3). (B) Effect of 30 µM PitStop2 (inhibits clathrin-dependent endocytosis). (C) Effect of 30 µM PitStop2 inactive (negative control). (D) Effect of 30 µM Dyngo4a (inhibitor of dynamin-dependent endocytosis). (E) Effect of 30 µM Dyngo4a inactive (negative control). (F) AUC calculated from A-D. For time course graphs, symbols show the mean and error

bars, standard error of the mean. The arrow indicates addition of vehicle or IPR agonist. Maximal FRET change induced by the positive control (forskolin and IBMX) is indicated as a dashed line. For AUC graphs, bars show the mean, symbols the individual data points, and error bars show the standard error of the mean. * p<0.05, ** p<0.01 and *** p<0.001 versus vehicle control, two-way ANOVA with Dunnett's multiple comparisons test.

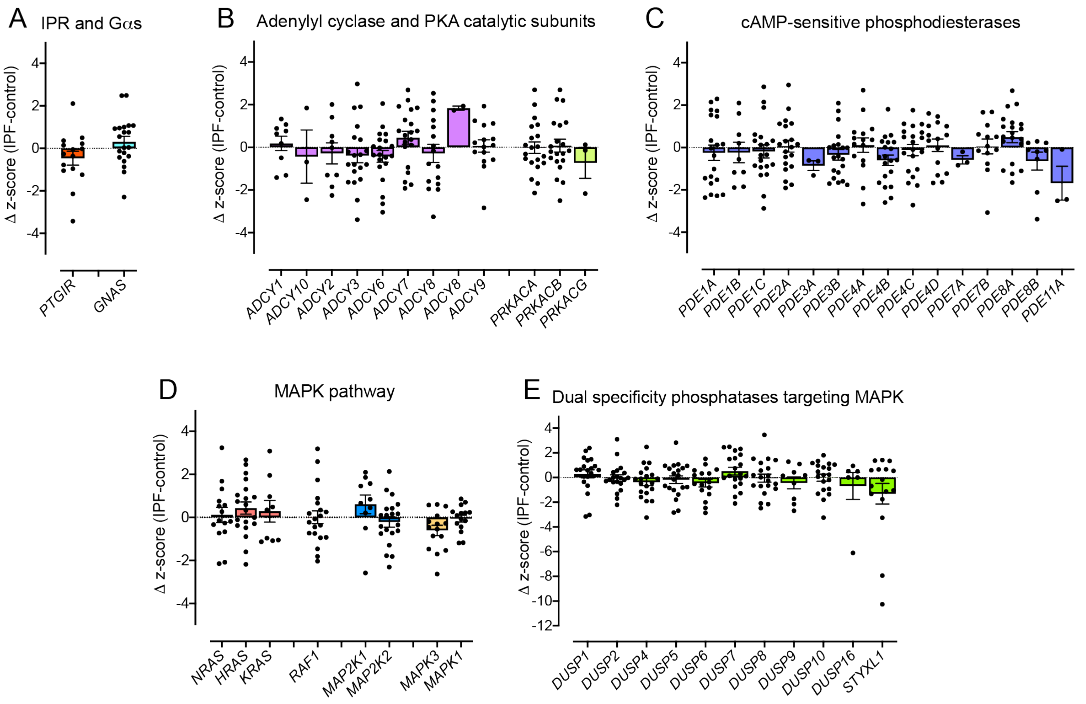

Supplementary Figure 5. Changes in gene expression from a meta-analysis of four studies comparing human lung fibroblasts from control vs IPF patients. We searched Supplementary Table 1 from (Plantier et al., 2016) for key signalling mediators that could influence the ability of IPR agonists to inhibit cell proliferation. (A) IPR receptor (*PTGIR*), Gαs (*GNAS*). (B) Adenylyl cyclase (AC) isoforms (*ADCY*) and PKA catalytic subunits (*PRKAC*). (C) cAMP-sensitive phosphodiesterases (*PDE*) (Omori and Kotera, 2007). (D) Key components of the MAPK pathway including Ras (*RAS*), Raf (*RAF1*), MEK (*MAP2K*) and ERK (*MAPK*). (E) Dual specificity phosphatases (*DUSP*) that target the MAPK pathway (Patterson et al., 2009). Data are expressed as z-score from IPF fibroblasts – z-score from control fibroblasts. Bars show the mean, symbols the individual data points, and error bars show the standard error of the mean.

Supplementary References

Omori, K., and Kotera, J. (2007). Overview of PDEs and their regulation. *Circ Res* 100**,** 309-327.

Patterson, K.I., Brummer, T., O'brien, P.M., and Daly, R.J. (2009). Dual-specificity phosphatases: critical regulators with diverse cellular targets. *Biochem J* 418**,** 475-489.

Plantier, L., Renaud, H., Respaud, R., Marchand-Adam, S., and Crestani, B. (2016). Transcriptome of Cultured Lung Fibroblasts in Idiopathic Pulmonary Fibrosis: Meta-Analysis of Publically Available Microarray Datasets Reveals Repression of Inflammation and Immunity Pathways. *Int J Mol Sci* 17.
